# Challenges in Differential Diagnosis of Diffuse Gingival Enlargement: Report of Two Representative Cases and Literature Review

**DOI:** 10.3390/dj12120403

**Published:** 2024-12-10

**Authors:** Erofili Papadopoulou, Maria Kouri, Anastasia Andreou, Smaragda Diamanti, Maria Georgaki, Konstantinos Katoumas, Spyridon Damaskos, Emmanouil Vardas, Evangelia Piperi, Nikolaos G. Nikitakis

**Affiliations:** 1 Department of Oral Medicine & Pathology and Hospital Dentistry, School of Dentistry, National and Kapodistrian University of Athens (NKUA), 11527 Athens, Greece; kourimari@yahoo.gr (M.K.); andreouanast@gmail.com (A.A.); margeor@dent.uoa.gr (M.G.); emvard@dent.uoa.gr (E.V.); liapiperi@dent.uoa.gr (E.P.); nnikitakis@dent.uoa.gr (N.G.N.); 2 Department of Dentistry, European University Cyprus, Nikosia 22006, Cyprus; 3 Department of Oral and Maxillofacial Surgery, School of Dentistry, National and Kapodistrian University of Athens (NKUA), 11527 Athens, Greece; kkatoumas@icloud.com; 4 Department of Oral Diagnosis and Radiology, School of Dentistry, National and Kapodistrian University of Athens (NKUA), 11527 Athens, Greece; sdamask@dent.uoa.gr

**Keywords:** diffuse gingival overgrowth, calcium channel blockers, diabetes mellitus, leukemia

## Abstract

**Background/Objectives:** The etiology of diffuse gingival enlargement is multifactorial, and the definitive diagnosis may be challenging. To highlight the nuances of the differential diagnosis, we present two cases of generalized gingival overgrowth and discuss the diagnostic dilemmas. **Case description:** In the first case, an 82-year-old male with a medical history of hypertension and prostatitis had a chief complaint of symptomatic oral lesions of a 20-day duration, accompanied by fever and loss of appetite. The clinical examination revealed diffusely enlarged, hemorrhagic, and focally ulcerative upper and lower gingiva, ecchymoses on the buccal mucosa, as well as bilateral cervical lymphadenitis. The histopathologic and immunohistochemical findings combined with the hematologic examination led to a final diagnosis of acute myeloid leukemia, and the patient was referred to a specialized hematology/oncology unit for further management. The second case was a 74-year-old female with a medical history of breast cancer (successfully managed in the past), type II diabetes mellitus, and cardiovascular disease, taking various medications. An intraoral examination revealed diffusely enlarged, erythematous, and hemorrhagic upper and lower gingiva. An incisional biopsy showed hyperplastic granulation and fibrous connective tissue with a predominantly chronic inflammatory infiltrate. Considering the patient’s medical history and current medications, the clinical and microscopic findings were in support of the diagnosis of drug-induced gingival overgrowth associated with calcium channel blocker (amlodipine), partially controlled diabetes serving as an additional predisposing factor. Gingivectomy and periodontal scaling, along with substitution of the offending medication, were curative, and better diabetic control was recommended. **Conclusions:** Diffuse gingival overgrowth may be caused by a variety of diverse conditions, ranging from an exuberant response to local factors, potentially exacerbated by hormonal influences (e.g., puberty or pregnancy), to drug side effects to genetic, systemic, or even neoplastic diseases. A careful evaluation of the medical and drug history and clinicopathologic correlation is essential for accurate diagnosis and appropriate management.

## 1. Introduction

Diffuse gingival enlargement or gingival overgrowth represents an excessive growth of the periodontal soft tissues, compared to their normal size, in a generalized distribution [1,2]. In general, the gingiva may undergo reactive proliferation driven by poor oral hygiene and accumulation of dental plaque and calculus, leading to inflammation (gingivitis), ensuing edema and granulation tissue formation [1]. Other local irritants, such as ill-fitting restorations, orthodontic devices, mouth breathing, and malocclusion, may worsen the clinical appearance [1,3]. Notwithstanding the fact that the majority of cases of gingival enlargement are due to the aforementioned local factors, a variety of other conditions, sometimes challenging both diagnosis- and management-wise, may also cause similar, possibly more pronounced clinical manifestations.

In terms of etiopathogenesis, gingival enlargement could be inflammation-related, medication-induced, associated with systemic and genetic diseases, or even of neoplastic nature [1,3]. It is noteworthy that the clinical presentation may be different depending on the causal factor. For instance, gingival enlargement may be more fibrotic, firm, and of normal color when it is drug-induced or associated with genetic syndromes, and more reddish, friable and prone to bleeding when related to inflammation, hormonal imbalances, and some systemic diseases [1,2,3]. Sometimes, gingival overgrowth may be the first manifestation of a systemic disease or even a malignancy and, thus, the first clue to their diagnosis [3]. Especially in cases associated with a malignant neoplasm, prompt diagnosis is essential as it leads to the early initiation of treatment, control of disease progression, and improvement in the patient’s prognosis and quality of life [1,2,3]. The differential diagnosis is challenging for the clinician and is based on patient’s medical, drug, and family history, as well as careful clinical examination, laboratory tests, and microscopic findings [3]. The aim of this study is to describe two cases of gingival overgrowth with different etiology, as representative examples of the range of possible implicating factors, and to discuss the diagnostic dilemmas that occur, while also providing a brief overview of the main underlying diseases and conditions that could manifest as diffuse gingival enlargement.

## 2. Case Reports

### 2.1. Case 1

An 82-year-old male presented with complaints about symptomatic oral lesions of a 20-day duration. The patient’s medical history was significant for hypertension and arrhythmia (on ramipril, hydrocholothiazide, and sotalol), hypercholesterolemia, prostatitis, and gastroesophageal reflux disease. He was a non-smoker.

**Figure 1:**
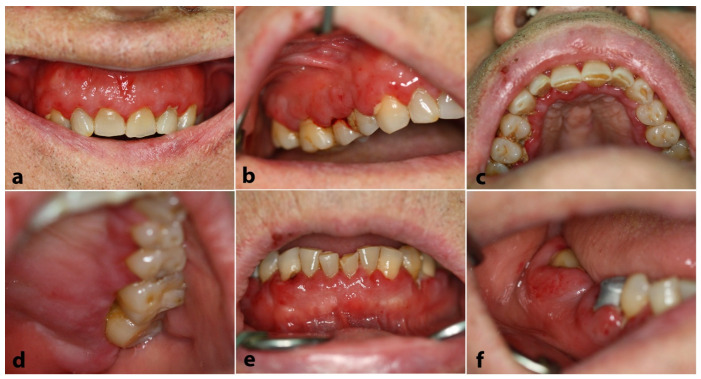
Case 1. Clinical examination: Diffuse enlargement, erythema with focal ulcerations, and high tendency for hemorrhage of the maxillary buccal (**a**,**b**) and anterior palatal (**c**) gingiva. Note the “tumor-like” mass located in the left hard palatal mucosa (**d**). Similar appearance of the lower anterior (**e**) and posterior (**f**) vestibular gingiva. A few teeth appeared to be almost covered by swollen gingiva (**f**).

The clinical examination revealed diffuse enlargement of the upper (Figure 1a–d) and lower gingiva (Figure 1e,f), which also appeared to be erythematous, hemorrhagic, and focally ulcerated.

**Figure 2:**
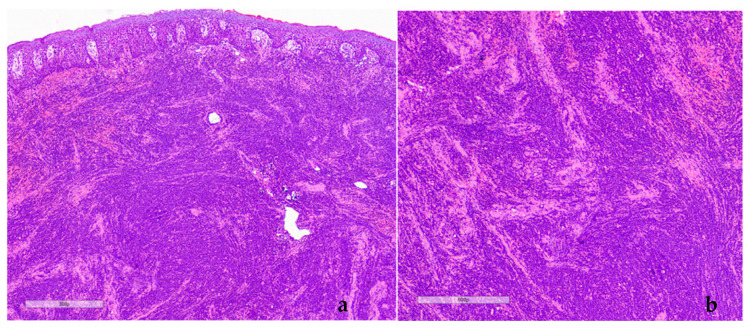
Case 1. Histopathologic evaluation (Hematoxylin and Eosin, initial magnification 200× (**a**) and magnification 400× (**b**)): Clusters of small- and medium-sized pleomorphic cells with hyperchromatic nuclei diffusely infiltrating the underlying connective tissue.

Additionally, there were ecchymoses on the buccal mucosa and bilateral cervical lymphadenitis. Due to the abrupt onset and severity of the condition, in the absence of related drug history, a provisional diagnosis of diffuse gingival enlargement due to systemic disease, possibly a hematologic malignancy, was set. Incisional biopsies from the left posterior palatal gingiva and the right posterior lower buccal gingiva were performed. Histopathologically, diffuse infiltration of the connective tissue by immature monocytic cells of small to medium size with hyperchromatic nuclei, with frequent atypical mitotic figures, was observed (Figure 2).

On immunohistochemical evaluation, the tumor cells strongly expressed CD33 and CD99, while the proliferation marker Ki-67 was positive in 80–90% of the neoplastic cells. The complete blood count (CBC) showed leukocytosis (white blood cell count: 70.66 k/μL; 95.7% lymphocytes), anemia (hematocrit: 36.0%), and thrombocytopenia (platelets: 22 k/μL), while inflammatory markers including C-reactive protein (CRP: 9.22 mg/dL) and erythrocyte sedimentation rate (ESR: 92 mm) were found to be elevated. A diagnosis of acute myelomonocytic leukemia (with gingival infiltration) was rendered, and based on further investigation via flow cytometry, a final diagnosis of acute myelomonocytic leukemia was established. The patient was referred to a specialized hematology/oncology unit for further evaluation and treatment; due to anemia and thrombocytopenia, red blood cell and platelet transfusion was performed while the patient was placed on antibiotics and antifungals (due to neutropenia and fever). Venetoclax, in combination with azacytidine, was the oncologic treatment of choice, as intensive chemotherapy was not opted for because of the patient’s advanced age.

### 2.2. Case 2

A 74-year-old female presented for the evaluation of asymptomatic gingival lesions (swelling), which she first noticed approximately two months prior. Her medical history was remarkable for diabetes mellitus (DM), which was reported as “partially controlled”, cardiovascular disease, and breast cancer (managed with surgery and chemotherapy 6 years prior; she had been receiving an aromatase inhibitor, which was discontinued 6 months prior). The patient was under gliclazide and metformin (antihyperglycemic agents), amlodipine [calcium channel blocker (CCB)], carvedilol (beta-blocker), amiodarone (antiarrythmic), and acenocoumarol (anticoagulant). She was a non-smoker.

**Figure 3:**
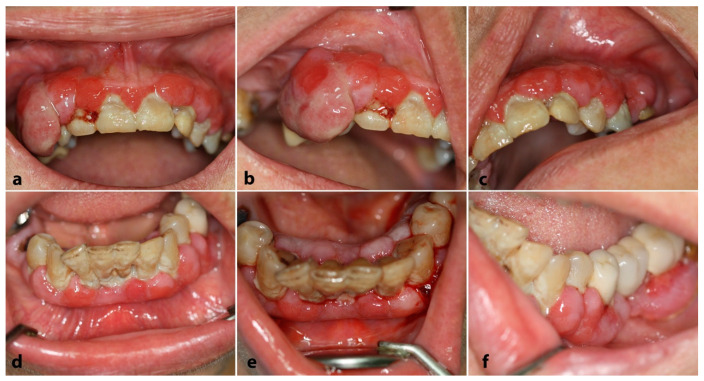
Case 2. Clinical examination: Diffusely enlarged, erythematous, and hemorrhagic upper (**a**–**c**) and lower (**d**–**f**) gingiva; multiple “pyogenic granuloma-like” lesions were also noticed.

On clinical examination, diffusely enlarged, erythematous, and hemorrhagic upper (Figure 3a–c) and lower (Figure 3d,e) gingiva were detected; in some areas, the gingival lesions were more pronounced, assuming the appearance of pyogenic granulomas (Figure 3a,b).

**Figure 4:**
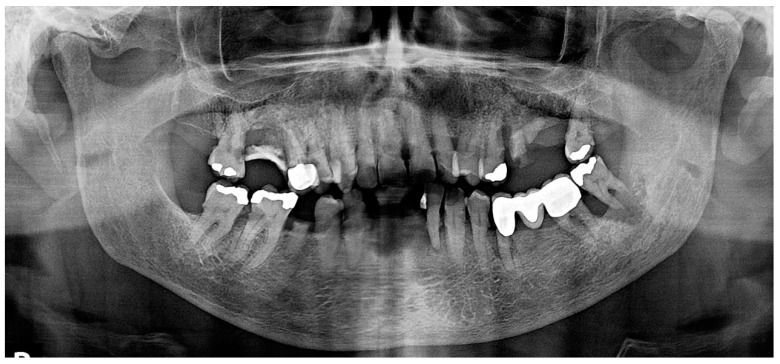
Case 2. Panoramic radiograph showing diffuse alveolar bone loss suggestive of advanced periodontitis. Note the radiographic evidence of calculus and the “floating in air tooth” appearance of the mandibular second left molar.

Panoramic radiography revealed multifocal alveolar bone loss consistent with advanced periodontal disease (Figure 4).

**Figure 5:**
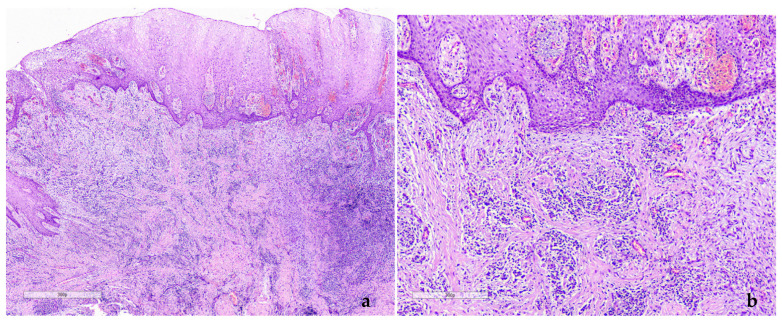
Case 2. Microscopic examination (Hematoxylin and Eosin, magnification 200× (**a**) and magnification 400× (**b**)): Areas of chronic inflammatory infiltrate and scattered variably sized vascular channels located in the lamina propria.

An incisional biopsy of the right anterior upper buccal gingiva and the left lower posterior buccal gingiva were performed and, on microscopy, hyperplastic granulation and fibrous connective tissue with dense mixed, mainly chronic, inflammatory infiltrate was noticed (Figure 5).

**Figure 6:**
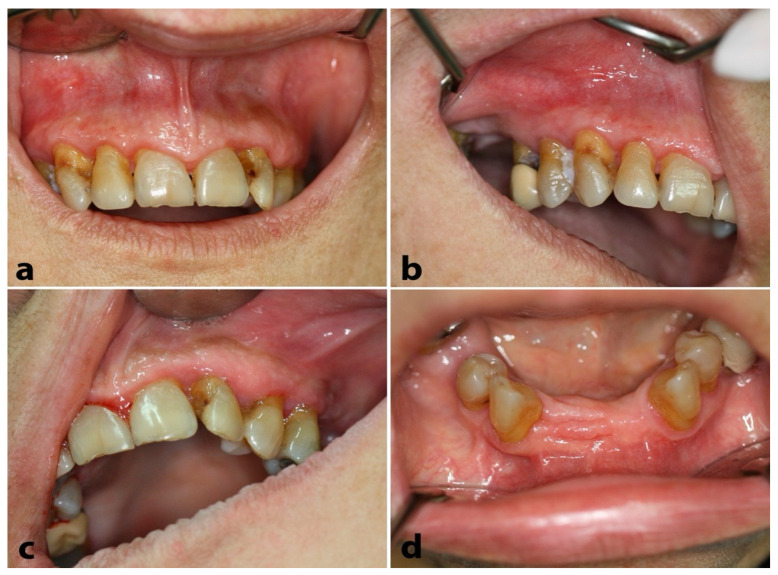
Case 2. Clinical re-evaluation 3 weeks after the completion of periodontal therapy, surgical excision of the hyperplastic gingival tissues, and extraction of teeth displaying terminal mobility: Complete healing and absence of gingival lesions in the upper (**a**–**c**) and lower (**d**) gingiva.

Taking into consideration the medical and drug history of the patient and, upon clinicopathologic correlation, the final diagnosis was drug-induced gingival overgrowth with focal pyogenic granuloma-like lesions, most likely related to CCB amlodipine; DM may also have served as an additional predisposing factor, especially as it was not adequately controlled (blood glucose was 141 mg/dL and HbA1C was 7.6%). At the same time, the poor oral hygiene status of the patient and the presence of dental plaque and calculus have obviously contributed and worsened the situation. Gingivectomy and periodontal treatment (root planning), as well as extraction of teeth exhibiting terminal mobility, were performed; at the same time, the responsible medication (amlodipine) was substituted with (non-CCB) candesartan and monoxidine, and dietary modifications for better glycemic control were implemented; the clinical outcome was quite satisfactory (Figure 6a–d).

## 3. Discussion

Gingival enlargement is a rather common clinical finding in cases of periodontal disease, including gingivitis and periodontitis, due to inflammation and granulation tissue formation. However, several conditions, such as hormonal imbalances (like those characterizing pregnancy and puberty), and various medications, as well as systemic or even neoplastic diseases, may also cause or contribute to the development of such a clinical appearance [3]. Hence, differential diagnosis is often a difficult and challenging task for the clinician, who should be familiar with all these entities and their nuances and follow the appropriate diagnostic methodology with the necessary investigations to reach the final diagnosis. Herein, we discuss entities that could manifest as diffuse gingival swelling with an emphasis on their clinicopathologic features and diagnostic work-up (summarized in Table 1).

Hyperplastic gingivitis/periodontitis (association with local factors, diabetes mellitus, and/or hormonal imbalances)

Hyperplastic gingivitis and periodontitis are inflammatory conditions caused by the deposition of microbial biofilm (plaque) and calculus on teeth; other local factors (e.g., malocclusion, tooth crowding, dental restorations with suboptimal margins, mouth breathing, and hyposalivation) may also contribute [4]. Inflammation is accompanied by edema, which, along with abundant granulation tissue formation and fibrosis, ensue in gingival enlargement. Furthermore, hormonal imbalances during adolescence and pregnancy, as well as suboptimally controlled DM, are major predisposing factors for hyperplastic periodontal disease [5]. Especially in pregnancy, increased levels of hormones in blood and saliva may trigger a periodontal inflammatory reaction and may also contribute to local reactive pyogenic granuloma-like growths, termed “pregnancy tumors” [6]. In cases of hyperplastic gingivitis, the gingival overgrowth may begin as a slight ballooning at the irritated sites that gradually increases in size and extent, leading to generalized enlargement. Frequently, the lesions are deep red and/or bluish, friable and soft, with a shiny surface, and bleed easily [4]. With time, the enlargement may become pink, firm, and fibrotic. Treatment consists of withdrawal of the irritant, i.e., plaque and calculus removal and oral hygiene measures, while surgical recontouring may be occasionally necessary; if there is no satisfactory response to local measures, other possible underlying systemic factors should be ruled out. In cases of DM, appropriate glycemic control, through medications and dietary modifications, is needed.

Drug-related gingival overgrowth

Several medications may cause diffuse gingival enlargement with consequent effects on oral hygiene, mastication, speech, and esthetics. Drug-induced gingival hyperplasia was first reported by Kimbell et al. in 1939 [7]. Medications related to the development of such lesions include calcineurin inhibitors (cyclosporine), CCBs (including nifedipine, amlodipine, diltiazem, and verapamil), anti-convulsants/anti-seizure drugs (mainly phenytoin), and oral contraceptives. The incidence of drug-related gingival overgrowth depends on the pharmacologic agent (higher for phenytoin), and the severity may be influenced by the patient’s genetic susceptibility and oral hygiene levels [4]. Usually, the condition appears 2–4 months after the initiation of the drug. The literature supports that lesions usually start from the interdental papilla and progressively involve marginal gingiva [3]. They may affect all sites of the gingiva, although they are more prominent in the anterior mandible and maxilla. Gingival enlargement may be localized or generalized, ranging from mild to severe with both esthetic and functional consequences [8]. When there is no prominent inflammation, lesions are pink, firm, lobulated, non-hemorrhagic, and painless. In cases of secondary inflammation (due to oral bacteria), lesions are reddish with a tendency to bleed. It has been reported that gingival enlargement caused by phenytoin is more fibrotic, while lesions caused by cyclosporine demonstrate more signs of inflammation [9]. It should be mentioned that the lesions do not commonly develop in edentulous regions and often improve after the extraction of the involved tooth [3]. Histopathologic features are non-pathognomonic, may resemble those of chronic hyperplastic periodontal disease, or even be consistent with pyogenic granuloma (like in the present Case 2). Furthermore, the responsible agent cannot be identified microscopically. The exact pathogenetic mechanism responsible for drug-related gingival enlargement remains unknown. A possible hypothesis for the pathogenesis of the lesion implicates an imbalance between cell proliferation and apoptosis resulting in hypercellularity [10]. Alternatively, it has been proposed that the responsible drugs decrease folate cellular uptake in gingival fibroblasts, finally resulting to an insufficient amount of active collagenase unable to breakdown excess gingival connective tissue [11]. Management includes improvement in oral hygiene and cessation of the responsible drug and substitution with another of a different class (in consultation with the attending physician) in order to prevent recurrence [8]. Surgical management for gingival recontouring using a scalpel or laser is frequently needed (as exemplified in Case 2).

Gingival fibromatosis

Gingival fibromatosis, first described by Gross in 1856 [12], is a rare, heterogenous group of disorders of unclarified etiopathogenesis, leading to the progressive growth of gingival tissue [13]. It is considered as a hereditary condition that can manifest either sporadically or in conjunction with various syndromes, including Hunter, Prune-Belly, Zimmerman–Laband, Rutherford, Byars–Jurkiewicz, Costello, Cross, and Ramon syndromes [14]. In rare cases, it is associated with syndromes leading to asymmetric overgrowth, as a result of true or partial facial hemihyperplasia, where one side of the face undergoes disproportionate development of all or part of its structures [15]. The onset more frequently coincides with the eruption of primary or permanent dentition. The lesions may worsen during adolescence, highlighting the potential role of hormones in disease progression. The disease develops slowly and appears as a local or diffuse non-bleeding enlargement within marginal and attached gingiva or interdental papilla. It usually does not extend beyond the muco-gingival junction. In the localized form, the most frequently affected areas are the gingiva (often palatal) of the upper molar region and the maxillary tuberosities [16]. In severe cases, the enlargement may cover the crowns of the involved teeth, causing functional, esthetic, and periodontal problems to the patients. Bone loss and bleeding due to biofilm accumulation may be noticed [13]. Histopathologic features are non-pathognomonic, showing an extremely collagenized hypocellular connective tissue [17]. The diagnosis is based on clinical features and medical and family history. Management is commonly surgical; more specifically, gingivectomy (with scalpel or laser) is usually suggested. Non-surgical management consists of scaling and root planning, oral hygiene measures, mouthrinses (e.g., chlorhexidine), and the administration of antibiotics, analgesics, and anti-inflammatory drugs. Regular follow-ups and efficient oral hygiene are thought to be mandatory in order to avoid relapses [13].

iLeukemia

Leukemia is a hematologic malignancy characterized by the excessive proliferation of immature white blood cells (WBCs) and their precursors. The proliferation of these malignant cells in the bone marrow (ΒΜ) results in the suppression of hematopoiesis, causing decreases in all functional blood cells [18]. All types of leukemia may give manifestations in the oral cavity, although more frequently so in acute types [18]. Leukemia’s oral complications may be due to tissue infiltration by malignant cells or the result of underlying anemia, thrombocytopenia, neutropenia, or impaired granulocyte function [19]. Similar to our first case, diffuse gingival enlargement, hemorrhage, petechiae and ecchymoses, and mucosal ulcers may be encountered. Interestingly, gingival involvement may be the initial presenting symptom in 5% of cases of acute myeloid leukemia [19]. Dreizen et al. reported that gingival involvement was frequent in patients with acute monocytic leukemia (66.7%), followed by acute myelomonocytic leukemia (18.5%) and acute myeloblastic leukemia (3.7%) [20]. The infiltration of gingival tissues by leukemic cells can lead to their generalized enlargement; at the same time, poor oral hygiene with plaque and calculus accumulation may deteriorate the condition causing gingival inflammation, edema and granulation tissue. Neutropenia and WBC dysfunction may also contribute to periodontal inflammation and manifest as ulcers, while bleeding, aggravated by thrombocytopenia, further complicate oral hygiene practice, causing a vicious cycle [21]. The enlarged tissues are usually soft, shiny, erythematous, and highly tender, usually bleeding on palpation. It is noteworthy that gingival infiltration is not commonly observed in edentulous areas, attesting to the importance of local factors [21]. The diagnosis of leukemia is suggested by CBC often showing significant or even dramatic increases in the total number of WBCs, in the presence of pancytopenia; blast cells are identified in the peripheral blood and BM [19]. A biopsy of affected gingiva reveals atypical cells with features reminiscent of myelomonocytes and/or lymphocytes, which express immunohistochemical markers indicative of their origin [18]. In addition, the role of molecular analysis in both the establishment of an accurate diagnosis as well as management with the selection of an appropriate targeted therapy is of high importance [22,23].

Granulomatous gingivitis

Granulomatous diseases include a variety of conditions of diverse etiology characterized histologically by the formation of granulomas. Oral manifestations may occur, sometimes preceding systemic signs and symptoms, raising diagnostic dilemmas for the clinician; in the case of the exclusive involvement of the orofacial tissues, the term orofacial granulomatosis is applicable [24]. Among other oral manifestations, granulomatous diseases may appear as gingival enlargement, also called granulomatous gingivitis. Granulomatous gingivitis may occur in cases of OFG and systemic diseases, such as tuberculosis, sarcoidosis, Crohn’s disease, and Wegener’s granulomatosis (the last of which is discussed in a separate section). Tuberculosis is a chronic infectious disease caused by Mycobacterium species and especially Mycobacterium tuberculosis and may be either primary or secondary [25]. Gingival occurrence of tuberculosis, especially primary tuberculosis, is rather rare [25,26]. Sarcoidosis is a granulomatous disease of unknown etiology, presenting more commonly with lung infiltration possibly affecting several organs; however, oral manifestations are rather uncommon [27,28]. Orofacial granulomatosis is a chronic inflammatory disease, first reported by Wiesenfeld et al. in 1985 [24]. The etiology of the disease is thought to be multifactorial, implicating genetic and hereditary factors, delayed hypersensitivity to allergens, microbial infections, and immunologic dysregulation [29,30]. The most common oral manifestation is lip swelling (granulomatous cheilitis), which is frequently combined with other oral clinical features (such as linear ulcerations, cobblestoning, mucosal tags, and a fissured tongue); the triad of granulomatous cheilitis, a fissured tongue, and facial palsy is termed Melkersson–Rosenthal syndrome [29,30]. The monitoring of OFG patients is needed, since many patients are later diagnosed with Crohn’s disease. The latter is an inflammatory disease that may affect any part of the gastrointestinal tract from the mouth to the anus; abdominal pain, nausea, and diarrhea are common, while oral manifestations may be indistinguishable from OFG; and there is a predisposition for aphthous ulcers and pyostomatitis vegetans [30,31].

In addition to the aforementioned possible etiologies, granulomatous gingivitis may be due to foreign body presence within the tissues. The source of the foreign material embedded within the gingiva is frequently iatrogenic (e.g., dental abrasives and restorative materials) or oral-hygiene-related (e.g., toothpaste). The clinical distribution may be localized or more widespread, appearing as erythematous swelling, sometimes accompanied by whitish or erosive areas [32,33].

The diagnosis of granulomatous gingivitis is confirmed by a biopsy and histopathologic examination, which reveal the presence of granulomas, composed of epitheliod histiocytes and multinucleated giant cells surrounded by lymphocytic inflammatory infiltrate. However, further investigation is needed in order to reach a final diagnosis. In cases of tuberculosis, microscopic features (i.e., caseous necrosis and Langhans giant cells) provide some diagnostic clues, but confirmation requires the identification of the organisms via special stains (such as Ziehl–Neelsen or other acid-fast stains), accompanied by other investigations, such as chest radiography, Mantoux test, culture, and nucleic acid amplification tests [26]. As far as sarcoidosis is concerned, the diagnosis is based on history, a lung investigation (chest radiography and pulmonary function tests), and hematologic and biochemical examinations (complete blood count, erythrocyte sedimentation rate, serum angiotensin-converting enzyme levels, and serum and urine calcium levels) [27,28]. The diagnosis of orofacial granulomatosis is made by the exclusion of the other granulomatous diseases, since there is no specific diagnostic test for it. In order to rule out Crohn’s disease, the patient should be referred to a gastrenterologist for the investigation of inflammatory bowel disease, which includes hematologic and stool tests, imaging, and endoscopy/colonoscopy [30,31]. Foreign body gingivitis is strongly suggested by the microscopic identification of polarizable foreign material within tissues in the appropriate clinicopathologic context [32,33].

Spongiotic gingival hyperplasia (SGH)

Localized gingival enlargements most often represent reactive lesions (such as pyogenic granuloma, peripheral fibroma, peripheral giant cell granuloma, and peripheral ossifying fibroma) related to traumatic factors, local irritations, or dental plaque accumulation. Another benign lesion that can present as localized gingival hyperplasia was first described by Darling et al. [34] as reddish gingival enlargements with spongiotic microscopic features affecting juveniles. Although the condition was initially referred to as localized juvenile spongiotic gingival hyperplasia (LJSGH), it may less often exhibit a multifocal distribution resembling other more diffuse gingival overgrowths. Therefore, the term spongiotic gingival hyperplasia (SGH) or spongiotic gingivitis has prevailed, considering that it is not always localized and does not affect only children and adolescents; indeed, more recent studies have noted that a considerable proportion of affected individuals are adults [35,36]. Another suggested term is spongiotic odontogenic gingivitis to account for its purported origin from odontogenic epithelium, specifically, the junctional or sulcular epithelium or odontogenic epithelial rests; noticeably, plaque is not considered to play a role in its pathogenesis [37,38]. Clinically, SGH is characterized by red, more often sessile lesions with a velvety or pebbly surface and soft consistency that can bleed easily, affecting the attached and marginal gingiva; a predilection for the upper anterior facial gingiva is seen [39]. The clinical presentation of SGH is often distinctive; however, it can be occasionally confused with other entities involving the gingiva as localized or more diffuse overgrowths. Biopsy and histopathologic examination are important in order to establish the final diagnosis. Microscopically, a papillary or pebbly surface, acanthosis, intercellular edema (spongiosis), exocytosis, and mixed inflammatory infiltrate are noticed; furthermore, in most cases, immunohistochemical evaluation for CK19 and CK8/18 is positive [37,38]. The condition is non-responsive to oral hygiene measures and appropriate treatment primarily involves conservative excision by a scalpel or laser, accompanied by regular follow-up, as recurrences may be observed [37].

Granulomatosis with polyangiitis—Wegener’s granulomatosis

Granulomatosis with polyangiitis (GPA), also termed Wegener’s granulomatosis in the past, was first described by Friedrich Wegener in 1936 [40] and represents a rare multiorgan disease of immunologic origin characterized by necrotizing granulomatous inflammation of the upper and lower respiratory tract, disseminated vasculitis, and glomerulonephritis [40,41]. There is a prevalence of 3 cases per 100.000 over a wide age range [40]. The disease may be limited or display an aggressive clinical course leading to the failure of multiple organs. There are two forms of GPA: (i) the localized form, mostly affecting the upper respiratory tract, sometimes limited to mucocutaneous surfaces only, and showing recurrences, and (ii) the disseminated form, a usually fatal disease with primarily renal and pulmonary involvement [41]. According to the American College of Rheumatology Criteria for the classification of GPA, two of the following criteria should be fulfilled for establishing the diagnosis: (a) oral ulcers or nasal discharge or inflammation, (b) nodules, fixed infiltrates, or cavities on a chest radiograph, (c) abnormal urinary sediment, and (d) granulomatous inflammation on biopsy material [40]. However, the revised classification criteria for GPA and their relative importance are as follows: blood nasal discharge, nasal crusting or sinonasal congestion (+3); cartilaginous involvement (+2); conductive or sensorineural hearing loss (+1); cytoplasmic antineutrophil cytoplasmic antibody (c-ANCA) or anti-proteinase 3 ANCA (PR3-ANCA) positivity (+5); pulmonary nodules, mass, or cavitation on chest imaging (+2); granuloma or giant cells on biopsy (+2); inflammation or consolidation of the nasal/paranasal sinuses or imaging (+1); pauci-immune glomerulonephritis (+1); perinuclear ANCA or antimyeloperoxidase ANCA positivity (−1); and eosinophil count ≥1 × 10^9^/L (−4). The patient is classified as having GPA if the cumulative score is ≥5 points [42]. Oral manifestations occur in 6–13% of cases (and may represent the first manifestation in a minority of patients), commonly characterize advanced GPA, and include erythematous gingival hyperplasia (reflecting the accumulation of granulomas), widely known as ‘strawberry gingivitis’ (due to a florid granular appearance); more rarely, mucosal ulcers (at any oral site) or other manifestations, e.g., palatal perforation or major salivary gland enlargement, may occur. Interestingly, oral lesions can be used as an indicator of disease progression [24,43,44,45], while granulomatous gingivitis may occur long before the development of disseminated GPA, sometimes being “the first to show” [46]. The lesions usually begin in the interdental papillae and extend to other areas of gingiva, resulting in periodontal attachment loss and tooth mobility, in addition to friable and hemorrhagic hyperplastic gingiva. Histopathologic findings include (1) the formation of granulomas with multinucleated giant cells, (2) foci of necrosis, and (3) leukocytoclastic vasculitis (perivascular mixed inflammatory infiltrate) [40]; importantly, as oral biopsy specimens rarely contain large blood vessels, the presence of vasculitis is not usually identifiable, and the features of granulomatous inflammation dominate. The detection of circulating ANCA (especially PR3-ANCA or c-ANCA; specificity of 80–100%) via indirect immunofluorescence and/or ELISA is another helpful diagnostic tool for GPA that could also monitor disease activity [47]. The treatment of choice is corticosteroids and cyclophosphamide, although the latter shows significant adverse events; therefore, other immunosuppressive regimens may be also administered [40].

Plasma cell gingivitis

Plasma cell gingivitis is a relatively uncommon disease, having been first described in the late 1960s. Although the exact etiopathogenesis of plasma cell gingivitis is not completely understood, it is thought to be a hypersensitivity reaction with affluent plasma cells [3,48]. The most commonly implicated allergens are toothpaste, chewing gums, food products, spices, and cinnamon. The disease may lead to severe gingival inflammation and mimic more serious conditions [49]. It affects all ages with a female predilection [48].

Diffuse gingival enlargement is present, and gingiva are bright red in color, with a loss of stippling, a slightly granular surface, and a tendency for bleeding, often accompanied by painful symptomatology; patients often complain about a burning sensation when eating hot or spicy food [3,48]. The disease typically shows a rapid onset and most frequently involves the free and attached gingiva, mainly in the maxillary and mandibular anterior segments. Extension into other sites, such as palatal and vestibular mucosa and the tongue, is possible; there are no signs of periodontal tissue involvement [50]. Histopathologic examination reveals a very dense inflammatory infiltrate of the lamina propria, primarily by plasma cells that exhibit polyclonality (ruling out plasmacytic neoplasms). Plasma cell gingivitis is a diagnosis of exclusion, based on careful history, clinicopathologic correlation, and hematologic examinations to exclude other entities. There is no universally effective treatment, but the identification and elimination of possible allergens usually leads to remission; however, the identification of the allergic culprit is challenging and may entail a detailed history, elimination diet, and/or allergy testing. Topical corticosteroids have been used to alleviate symptoms, while systemic steroids, antibiotics, and immunomodulators may be employed in persistent cases [48,51]. Oral hygiene measures may also be helpful, especially plaque-control mouthwashes for removing local irritants and reducing immunological cell-mediated and cytokine-mediated responses [50]. However, recurrences are common.

Kaposi sarcoma

Kaposi sarcoma is a neoplasm of endothelial origin. It was first described by Moritz Kaposi in 1872 as several cases of multifocal pigmented sarcomas of the skin [52]. Only in 1994 was it found that the disease is of infectious origin, and Herpersvirus-8 (HHV-8 or Kaposi Sarcoma-associated Virus (KSV)) was recognized as the cause [53]. There are four epidemiologic subtypes: classic (or sporadic); endemic, found in Africa; AIDS-related; and iatrogenic, the last of which is reported in patients with iatrogenic immunodeficiency, such as organ transplant recipients [52]. Oral manifestations are common in the AIDS-related form, being present in approximately 60–80% of patients with KS and sometimes constituting the first site of disease appearance [54]. However, the incidence of the tumor has declined following the implementation of highly active antiretroviral therapy (HAART) [54]. The clinical presentation of oral Kaposi sarcoma may vary from single spots to ulcerative, painful swellings, mainly on the palate and gingiva. Kaposi sarcoma may initially appear as macules, progressively developing into plaques and extensive nodules on the gingiva, with their color ranging from pink to red or vivid purple; they may cause dysphagia and tooth mobility (due to alveolar bone involvement) [52]. Their diagnosis should be confirmed histologically [55]. Microscopic findings for Kaposi sarcoma include an increased number of vessels, extravasated red blood cells, and the proliferation of pleomorphic spindle cells in sheets or fascicles forming slit-like vascular spaces; three stages of development (patch, plaque, and nodular) are recognized [55,56]. Immunohistochemistry is essential for the final diagnosis, including CD34 (showing the vascular origin of the cells), VEGF3 and LYVE1 (showing the lymphatic origin), as well as LANA protein (indicative of the HHV-8) [52]. Treatment options include topical therapies with 9-cis-retinoid acid (alitretinoin gel 0.1%) or imiquimod cream 5%, intralesional chemotherapies (vinblastine, vincristine, and bleomycin), electrochemotherapy (a combination of intralesional chemotherapy and electroporation), and radiotherapy using low-energy photons [57], the last of which can lead to severe mucositis. Surgical excision has been employed but is associated with a high recurrence rate, while cryotherapy and laser (CO_2_) therapy are additional alternatives [57]. Systemic treatment strategies are variable, including doxorubicin, paclitaxel, anthracyclines, taxanes, m-TOR inhibitors, and interferon alpha, depending upon the type of KS, disease extent, and patient’s symptoms; for AIDS patients, HAART can induce regression and, depending on the stage, may be administered alone or in combination with other systemic therapies [57,58].

Scurvy

Vitamin C is important for collagen formation and maturation and for the maintenance of the basement membrane, also playing an equally important role in iron absorption; its deficiency results in vascular and tissue fragility and poor wound healing [59,60]. Nowadays, hypovitaminosis C, although much less frequent today in comparison with the past, still exists. In developed countries, scurvy could appear in elderly individuals with a diet poor in fresh fruits and vegetables, alcoholics, patients with mental illness, or even infants whose diet consists entirely of milk [60]. According to the World Health Organization (WHO), the diagnosis of scurvy is confirmed when serum ascorbic levels are less than 2 μg/mL [3,59]. Vitamin C deficiency usually develops 1–3 months after inadequate citrus, fruit, and/or vegetable intake [60]. “Scorbutic gingivitis” is the most common oral manifestation of scurvy, characterized by intensely reddish, hemorrhagic, and painful gingiva that exhibit generalized swelling [61]. In cases of severe vitamin C deficiency, gingiva may develop ulcerations, while significant involvement of the periodontal tissues with alveolar bone loss and tooth mobility may be observed. Oral hygiene is usually poor, and gingival overgrowth is often accompanied by malodor [59]. Hemorrhagic tendency with petechiae and ecchymoses may be noticed in other oral mucosa sites [62]. Replacement therapy is the only intervention required in such cases.

## 4. Conclusions

Diffuse enlargement of the gingiva is a relatively common manifestation of gingivitis or periodontitis. Nevertheless, several drugs, such as calcium channel blockers (as in our second case), may cause a similar clinical appearance, which may be exacerbated by the presence of dental plaque. In addition, many other systemic conditions, such as uncontrolled DM (as in our second case) and hematologic malignancies, most importantly leukemia (as in our first case), may cause similar gingival overgrowth. Careful evaluation and clinicopathologic correlation are required for accurate diagnosis and appropriate management; patients’ education on oral hygiene and regular dental examination is also necessary.

## Figures and Tables

**Table 1:** Differential diagnosis of entities manifesting as diffuse gingival enlargement.

Entity	Biofilm Correlation	Medical and Drug History	Clinical Findings	Microscopic Features	Diagnostic Procedure	Treatment Options
Hyperplastic gingivitis/periodontitis	Yes (strong)	Non-contributory (or co-existence of contributing factors)	Enlarged, erythematous and hemorrhagic gingiva, periodontal pockets (in periodontitis)	Non-pathognomonic; chronic or mixed inflammatory infiltrate; abundant granulation tissue and fibrosis	Clinical and radiographic evaluation	Periodontal treatment and oral hygiene improvement; surgical recontouring (if necessary)
DM-associated hyperplastic gingivitis/periodontitis	Yes	DM (poorly controlled; elevated blood glucose and/or HbA1C levels)	Same as above (other oral manifestations of DM may co-exist, e.g., candidiasis, xerostomia, RAS)	Same as above	Clinical and radiographic evaluation; appropriate laboratory tests for DM assessment	Periodontal treatment and oral hygiene improvement; pharmacologic and/or dietary management of DM
Hormonal imbalance-associated hyperplastic gingivitis/periodontitis	Yes	Puberty, pregnancy	Same as above (pyogenic granuloma-like lesions may occur, especially in pregnancy, i.e., “pregnancy tumors”)	Same as above	Clinical and radiographic evaluation	Periodontal treatment and oral hygiene improvement
Drug-related gingival overgrowth	Yes (variable)	Anti-convulsants, CCBs, cyclosporin, oral contraceptives	Enlarged gingiva of erythematous, hemorrhagic, and/or firm/fibrous consistency; may occasionally resemble pyogenic granulomas	Non-specific; hyperplastic fibrous connective and/or granulation tissue, chronic inflammation of variable degree	Medical history, clinical and radiographic examination	Periodontal treatment and oral hygiene improvement, gingivectomy, discontinuation or replacement of medication
Gingival fibromatosis	No	Hereditary, onset in childhood; hypertrichosis, epilepsy, intellectual disability, hypothyroidism, clondrodystrophia, growth hormone deficiency	Bilateral and symmetrical gingival enlargement; gingiva are of normal hue	Hyperplastic densely collagenized fibrous connective tissue with scattered fibroblasts and absent or mild inflammation	Medical and family history, clinicopathologic correlation	Surgical excision, oral hygiene improvement, periodontal treatment
Leukemia (with gingival infiltrate)	Weak	“B-symptoms” may be present	Gingiva usually described as “boggy” and hemorrhagic; ulcers, petechiae, and/or ecchymoses may co-exist	Tissue infiltration by atypical cells with myelomonocytic or lymphoid characteristics	CBC, BM biopsy, gingival biopsy and histopathologic examination, IHC and molecular analysis	Chemotherapy, targeted agents, BM transplantation
Granulomatous gingivitis	Very weak	Non-contributory in OFG and foreign body-related; possible history, signs, and symptoms of systemic granulomatous diseases (Crohn’s disease, tuberculosis, sarcoidosis)	Enlarged, erythematous gingiva; persistent, possibly symptomatic; other possible oral manifestations (swollen lips, cobblestone mucosal appearance, mucosal tags, linear ulcerations, fissured tongue)	Granulomas with epithelioid histiocytes and multinucleated giant cells (caseous necrosis in tuberculosis, polarizable foreign material in foreign body gingivitis)	Medical history, biopsy and histopathologic examination (special stains), specific work-up (e.g., chest X-ray, culture, endoscopy/colonoscopy, other laboratory tests)	Topical, intralesional, or systemic corticosteroids, other immunosuppressants; elimination diet (for OFG or Crohn’s); surgical recontouring (if there is no response to medications); management of the underlying systemic condition
Spongiotic gingival hyperplasia	No	None	Red sessile lesions with velvet or pebbly surface and soft consistency	Lack of keratinization, spongiosis, exocytosis, mixed inflammatory infiltration	Clinicopathologic correlation, IHC evaluation (CK19 and CK8/18)	Surgical excision, recurrences may occur (no response to oral hygiene measures)
Granulomatosis with polyangiitis (Wegener’s granulomatosis)	No	Sinonasal signs and symptoms; renal and respiratory tract involvement	“Strawberry-like” gingivitis, oral ulcerations may be also present	Granulomatous inflammation including multinucleated giant cells, necrosis, and leukocytoclastic vasculitis (rarely seen in oral biopsy material)	Clinicopathologic correlation, radiographic evaluation (chest, sinus), laboratory tests (including PR3-ANCA or c-ANCA)	Corticosteroids, cyclophosphamide, and/or other immunosuppressants
Plasma cell gingivitis	Weak	Variable allergens (e.g., chewing gums, toothpaste, mouthwashes, spices); idiopathic	Rapid onset, enlarged brightly erythematous gingiva with loss of normal stippling, possibly symptomatic (burning sensation)	Dense inflammatory infiltrate composed of plasma cells (polyclonal)	Clinicopathologic correlation, IHC evaluation (κ or λ light chains and CD138)	Modification of diet and/or oral hygiene products, topical or systemic immunosuppressive medications
Kaposi sarcoma	No	Immunosuppression, especially in HIV infection	Purplish-blue gingival enlargement, hemorrhagic	Proliferation of pleomorphic spindle cells forming slit-like vascular spaces	Clinicopathologic correlation, IHC evaluation, medical work-up	Chemotherapy (intralesional or systemic), topical therapies, surgical excision
Scurvy	Mild	Diet lacking fruits and vegetables, infants only feeding on milk, older men	Enlarged, erythematous, hemorrhagic, and fragile gingiva; tooth mobility; mucosal petechiae and ecchymoses	Non-specific	Vitamin C deficiency upon biochemical analysis	Replacement therapy

Abbreviations: DM: diabetes mellitus; RAS: recurrent aphthous stomatitis; CCB: calcium channel blockers; CBC: complete blood count; BM: bone marrow biopsy; IHC: immunohistochemistry; OFG: orofacial granulomatosis; ANCA: antineutrophil cytoplasmic antibody.

## Data Availability

The data presented in this study are available on request from the corresponding author on reasonable request. The data are not publicly available due to privacy policies.
